# Structure-antioxidant activity relationship of methoxy, phenolic hydroxyl, and carboxylic acid groups of phenolic acids

**DOI:** 10.1038/s41598-020-59451-z

**Published:** 2020-02-13

**Authors:** Jinxiang Chen, Jing Yang, Lanlan Ma, Jun Li, Nasir Shahzad, Chan Kyung Kim

**Affiliations:** 1 School of Chemical Engineering and Technology, North University of China, Taiyuan, 030051 China; 2 Department of Chemistry and Chemical Engineering, Inha University, Incheon, 22212 Korea

**Keywords:** Computational chemistry, Computational chemistry, Small molecules, Small molecules

## Abstract

The antioxidant activities of 18 typical phenolic acids were investigated using 2, 2′-diphenyl-1-picrylhydrazyl (DPPH) and ferric ion reducing antioxidant power (FRAP) assays. Five thermodynamic parameters involving hydrogen atom transfer (HAT), single-electron transfer followed by proton transfer (SET-PT), and sequential proton-loss electron transfer (SPLET) mechanisms were calculated using density functional theory with the B3LYP/UB3LYP functional and 6–311++G (d, p) basis set and compared in the phenolic acids. Based on the same substituents on the benzene ring, -CH_2_COOH and -CH = CHCOOH can enhance the antioxidant activities of phenolic acids, compared with -COOH. Methoxyl (-OCH_3_) and phenolic hydroxyl (-OH) groups can also promote the antioxidant activities of phenolic acids. These results relate to the O-H bond dissociation enthalpy of the phenolic hydroxyl group in phenolic acids and the values of proton affinity and electron transfer enthalpy (ETE) involved in the electron donation ability of functional groups. In addition, we speculated that HAT, SET-PT, and SPLET mechanisms may occur in the DPPH reaction system. Whereas SPLET was the main reaction mechanism in the FRAP system, because, except for 4-hydroxyphenyl acid, the ETE values of the phenolic acids in water were consistent with the experimental results.

## Introduction

Phenolic acids, a class of compounds formed by the substitution of hydrogen atoms on benzene rings by a carboxylic acid group and at least one hydroxyl, are widely found in plants, plant foods, and human metabolites^1^. Unlike flavonoids, free phenolic acids, such as benzoic, phenylacetic, and cinnamic acids, have high bioavailability and good water solubility^2^. They can be absorbed in the stomach, whereas flavonoids cannot be absorbed, and only a small amount of flavonoids are transported passively through the intestinal wall into the blood^3–5^. Most flavonoids are affected by pH, and digestive enzymes and intestinal microorganisms jointly affect C-ring cleavage, which breaks down into phenolic acids before being absorbed into the blood circulation system^6–8^. Like flavonoids, phenolic acids are considered to be excellent antioxidants that can quench excessive free radical-induced body damage and chronic diseases^2^. The antioxidant ability center of phenolic acids is phenolic hydroxyl, so the number and position of phenolic hydroxyls are directly related to their antioxidant activity^9^. Moreover, the methoxy and carboxylic acid groups also have important effects on the antioxidant ability of phenolic acids^10,11^.

In recent years, with the development of computational chemistry based on density functional theory (DFT), theoretical results are often used to further explain the experimental results or predict the antioxidant activity of phenolic acids^12^. Three key antioxidant mechanisms involved in the process of quenching free radicals are hydrogen atom transfer (HAT), single-electron transfer followed by proton transfer (SET-PT), and sequential proton-loss electron transfer (SPLET). HAT is a one-step reaction related to O-H bond dissociation enthalpy (BDE), whereas SET-PT and SPLET are two-step reactions, the former is related to ionization potential (IP) and proton dissociation enthalpy (PDE), and the latter is related to proton affinity (PA) and electron transfer enthalpy (ETE)^13,14^. These reaction mechanisms under different micro-environments may occur independently or simultaneously at different rates^15^.

In this study, to elucidate the structure-activity relationships (SAR) of phenolic antioxidants, 18 typical phenolic acids, hydroxybenzoic acid (6), hydroxyphenylacetic acid (6), and hydroxycinnamic acid (6), from natural products or/and colon metabolites of polyphenols with corresponding structures, were investigated by experimental and computational methods^16^. Their antioxidant activities were evaluated using a 2,2′-diphenyl-1-picrylhydrazyl (DPPH) assay in an ethanol system and ferric ion reducing antioxidant power (FRAP) assay in a water system. Here, five thermodynamic parameters of phenolic acids, BDE, IP, PDE, PA, and ETE, were calculated under three different micro-environments (ethanol, water, and gas) at the B3LYP/6–311++G (d, p)//UB3LYP/6-311++G (d, p) level. Moreover, the energy of the highest occupied molecular orbital (HOMO) in the three micro-environments was also computed to better describe the radical scavenging reactivity of the studied compounds. Finally, the effects of the methoxy, phenolic hydroxyl, and carboxylic acid groups on the antioxidant activity of phenolic acids and the possible mechanism of these effects will be discussed.

## Results and Discussion

### Experimental study of phenolic acids

**Figure 1:**
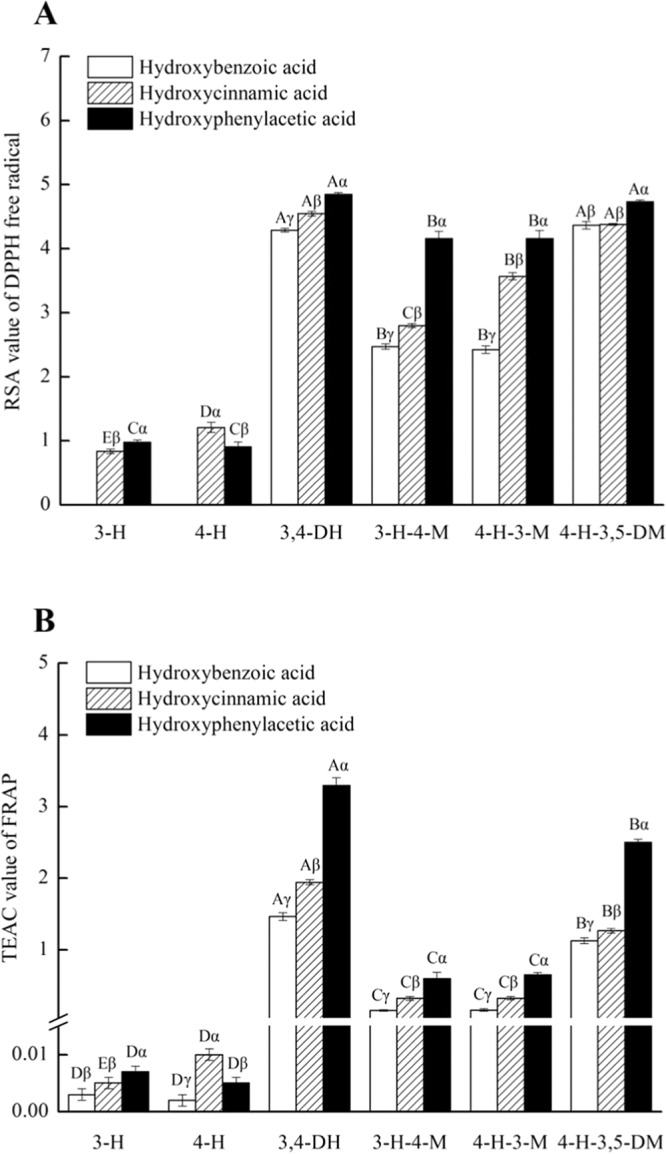
RSA values of DPPH (**A**) and TEAC value of FRAP (**B**) in 18 tested compounds. The data were expressed as mean (±SD) (n = 3). Different lowercase Greek letters represented different phenolic acids with the same methoxy and phenolic hydroxyl groups (*p*  < 0.05), and different Latin letters represented different phenolic acids with the same carboxylic acid group (*p* < 0.05).

Phenolic acids are considered excellent natural antioxidants, which have potential applications in medicine and health food. In this study, the antioxidant activity of 18 phenolic acids is expressed by the radical scavenging activity (RSA) value of scavenging DPPH^•^ and the trolox equivalent antioxidant capacity (TEAC) value of the FRAP method (Fig. 1 and Table S1). The names of the phenolic acids and abbreviations are shown in Table 1.

**Table 1:** Molecular structures of hydroxybenzoic, hydroxyphenylacetic and hydroxycinnamic acids.

Basic structures	Abbreviations	Compounds	Substituents		
			R_1_	R_2_	R_3_
	3-H-B	3-Hydroxybenzoic acid	OH	H	H
	4-H-B	4-Hydroxybenzoic acid	H	OH	H
	3,4-DH-B	Protocatechuic acid	OH	OH	H
	3-H-4-M-B	Isovanillic acid	OH	OCH_3_	H
	4-H-3-M-B	Vanillic acid	OCH_3_	OH	H
	4-H-3,5-DM-B	Syringic acid	OCH_3_	OH	OCH_3_
	3-H-C	3-Hydroxycinnamic acid	OH	H	H
	4-H-C	*p*-Coumaric acid	H	OH	H
	3,4-DH-C	Caffeic acid	OH	OH	H
	3-H-4-M-C	Isoferulic acid	OH	OCH_3_	H
	4-H-3-M-C	Ferulic acid	OCH_3_	OH	H
	4-H-3,5-DM-C	Sinapic acid	OCH_3_	OH	OCH_3_
	3-H-P	3-Hydroxyphenylacetic acid	OH	H	H
	4-H-P	4-Hydroxyphenylacetic acid	H	OH	H
	3,4-DH-P	3,4-Dihydroxyphenylacetic acid	OH	OH	H
	3-H-4-M-P	Homoisovanillic acid	OH	OCH_3_	H
	4-H-3-M-P	Homovanillic acid	OCH_3_	OH	H
	4-H-3,5-DM-P	4-Hydroxy-3,5-dimethoxyphenylacetic acid	OCH_3_	OH	OCH_3_

#### Effect of the carboxylic acid group on antioxidant activity

As shown in Fig. 1, when the other substituents of the benzene ring were the same, the trend in the antioxidant activity of three different phenolic acids in the two detection systems was as follows: hydroxyphenylacetic acid (-CH_2_COOH) > hydroxycinnamic acid (-CH = CHCOOH) > hydroxybenzoic acid (-COOH). Similarly, Natella *et al*. reported that hydroxycinnamic acid had stronger antioxidant activity than hydroxybenzoic acid when other substituents of benzene ring were the same^17^. Siquet *et al*. also reported that 3,4-dihydroxyphenylacetic acid (3,4-DH-P) had stronger antioxidant activity than caffeic acid (3,4-DH-C) and protocatechuic acid (3,4-DH-B)^11^. These results may be related to the electron-donating ability of carboxylic acid groups. The conjugation effect and induction effect together determine that -COOH is a strong electron-withdrawing group, -CH = CHCOOH is a weak electron-withdrawing group, and -CH_2_COOH is a weak electron-donating group. An electron-donating group can increase the electron cloud density of the benzene ring, decrease the dissociation energy of the phenolic hydroxyl bond and then enhance its free radical scavenging ability. For example, -NO_2_ is considered to be a strong electron-withdrawing group that enhances the dissociation energy of the -OH bond of 3,5-dinitrosalicylic acid, which is about 10 kcal/mol higher than that of 3-methoxysalicylic acid. Similarly, the antioxidant activity of the former is lower^18^. Therefore, we speculate that the carboxylic acid groups affect the antioxidant activity of phenolic acids according to their electron-donating ability (-CH_2_COOH > -CH = CHCOOH > -COOH).

However, the reaction systems may interfere with the above rules. Both ABTS and FRAP assays react in a water system, whereas DPPH reacts in an ethanol system. In a study of six dihydrochalcone compounds in *Malus*, the antioxidant activity of phlorizin was found to be the lowest in a DPPH assay, whereas the antioxidant activity of sieboldin was the lowest in an ABTS assay^19^. In the ethanol system, 4-H-3-M-C is more conducive to scavenging free radicals^20^. In this study, there is no significant difference in antioxidant activity between the syringic acid (4-H-3,5-DM-B) of the benzoic acid group and the sinapic acid (4-H-3,5-DM-C) of the cinnamic acid group in the DPPH assay (*P* > 0.05), whereas the former is higher than the latter in the FRAP assay. This may be related to the formation of intramolecular hydrogen bonds between the 4-OH and *o*-methoxy groups^10^. Intermolecular hydrogen bonds between the sinapic acid and ethanol solvent can reduce the role of intramolecular hydrogen bonds, and the polarity of ethanol may not be great enough to completely offset the intramolecular hydrogen bonds formed by the phenolic hydroxyl and *o*-methoxy in sinapic acid, so it exhibits a relatively lower antioxidant activity in the DPPH assay compared with the FRAP assay (Fig. 1). Therefore, the effect of the reaction system should be considered when determining the antioxidant activity of compounds.

#### Effect of the methoxy and phenolic hydroxyl groups on antioxidant activity

Under the same mother nucleus structure, the more the number of the methoxyl groups, the higher is the antioxidant activity of phenolic acids. The basic rules are as follows: 4-H-3,5-DM > 4-H-3-M > 3-H-4-M > 4-H > 3-H in the DPPH and FRAP assays (Fig. 1). Similarly, it was reported that the antioxidant activity of 4-H-3,5-DM-B was significantly stronger than that of 4-H-3-M-B, 3-H-4-M-B, and 4-H-B^10^. Moreover, the methoxyl group not only affects the antioxidant activity of phenolic acids but also enhances stilbenes, flavonoids, and hydroxytyrosol, which have conjugated systems^21,22^. It is worth mentioning that in the benzoic acid group, the RSA_DPPH_ value of 4-H-3,5-DM-B is more than 4 times higher than that of 4-H-B (Table S1), which means that their ability to scavenge free radicals differs by tens of thousands of times, and the methoxyl group greatly improves the antioxidant activity of phenolic acids.

In phenolic acids the number and position of phenolic hydroxyl groups are directly related to the free radical scavenging ability^11^. When the number of phenolic hydroxyl groups on the benzene ring is less than 4, the antioxidant activity of phenolic acids is proportional to the number of phenolic hydroxyl groups^10^. Moreover, because phenolic hydroxyl groups are electron donor groups they can enhance the antioxidant activity of other phenolic hydroxyl^23^. In this study, dihydroxy phenolic acids (3,4-DH) had a higher antioxidant activity than other phenolic acids with corresponding carboxylic acid groups in FRAP and DPPH assays apart from 4-H-3,5-DM-B/C/P.

In general, both phenolic hydroxyl and methoxy groups significantly enhance the antioxidant activity of phenolic acids.

### Computational study of phenolic acids

To gain further insights into the SAR of phenolic acids, we investigated the mechanistic pathway of the antioxidant activity on the basis of thermodynamic parameters. Previous studies have shown that hydroxyphenol is the antioxidant activity center, and its hydrogen-donating ability is affected by the polarity of the solvent. Moreover, the experimental studies are conducted with an ethanol system (DPPH assay) and a water system (FRAP assay). Here, ethanol or water, and gas (an extreme condition) are used as the micro-environments to calculate the thermodynamic parameters.

#### HAT mechanism

It is clear that the BDE is an important parameter in relation to the HAT mechanism. The lower the BDE value, the lower is the stability of the corresponding OH bond, which indicates that the OH bond is easily broken. The calculated OH BDEs of phenolic acids have a similar order in the three micro-environments (Table 2). When the substitution positions of the methoxy and phenolic hydroxyl groups on the benzene ring are the same, the BDE values in hydroxyphenylacetic acid (P) and hydroxycinnamic acid (C) are 1.9–13.3 kcal/mol and 0.9–9.2 kcal/mol lower than the corresponding BDE values of hydroxybenzoic acid (B), respectively. This shows that -CH_2_COOH can decrease the dissociation energy of the phenolic hydroxyl bond, thereby enhancing free radical scavenging ability, which is consistent with the experimental results above. Hydroxybenzoic acid and hydroxycinnamic acid have a stronger antioxidant activity than the corresponding hydroxybenzoic acid in the DPPH and FRAP assays.

**Table 2:** The calculated thermodynamic parameters of 18 tested compounds in gas and solvents at the B3LYP/6-31++G(d,p) level.

Compounds	Bonds	BDE (kcal·mol^−1^)			IP (kcal·mol^−1^)			PDE (kcal·mol^−1^)			PA (kcal·mol^−1^)			ETE (kcal·mol^−1^)		
		gas	ethanol	water	gas	ethanol	water	gas	ethanol	water	gas	ethanol	water	gas	ethanol	water
3-H-B	3-OH	85.9	87.4	85.5	197.1	126.9	119.3	202.5	7.0	13.3	339.8	42.3	45.5	61.3	91.6	87.2
3-H-C	3-OH	84.7	86.8	82.9	189.3	123.2	114.5	209.1	10.1	15.6	336.1	43.4	45.4	63.9	89.9	84.6
3-H-P	3-OH	83.7	85.2	82.4	189.3	121.1	113.0	208.1	10.6	16.6	342.2	44.6	46.6	56.7	87.1	83.0
4-H-B	4-OH	86.2	88.9	86.7	198.1	129.2	120.6	201.8	6.2	13.3	331.5	38.9	41.5	69.9	96.6	92.4
4-H-C	4-OH	82.4	83.8	81.4	182.9	118.1	110.1	213.2	12.2	18.4	326.7	39.1	41.9	69.5	91.2	86.7
4-H-P	4-OH	83.7	83.9	81.5	186.8	118.9	111.0	210.6	11.5	17.6	342.6	44.6	46.7	56.3	85.8	82.0
3,4-DH-B	3-OH	85.0	83.3	80.9	188.8	120.5	112.6	209.8	9.3	15.5	342.9	42.9	44.6	57.3	87.0	83.5
	4-OH	76.6	81.3	79.2				201.4	7.3	13.7	323.4	35.2	38.1	68.4	92.6	88.3
3,4-DH-C	3-OH	84.6	82.2	80.1	179.8	114.4	107.0	218.5	14.4	20.3	339.2	41.3	48.0	49.3	87.4	79.3
	4-OH	73.3	77.3	75.7				207.2	9.4	15.9	319.4	33.9	40.1	80.4	89.8	82.7
3,4-DH-P	3-OH	83.8	80.5	77.9	180.2	113.2	105.4	217.3	13.8	19.7	346.4	44.7	46.4	52.5	82.3	78.6
	4-OH	74.4	77.2	75.0				207.9	10.5	16.8	332.8	40.6	43.1	56.9	83.1	79.1
3-H-4-M-B	3-OH	85.0	83.3	80.8	185.1	118.8	110.9	214.9	11.0	17.0	344.5	43.7	45.3	55.8	86.1	82.7
3-H-4-M-C	3-OH	84.1	81.6	78.9	175.2	112.5	104.7	223.5	15.5	21.4	340.9	44.0	45.6	58.4	84.1	80.4
3-H-4-M-P	3-OH	83.0	80.1	77.4	175.3	112.5	103.5	221.4	14.1	21.0	347.4	45.4	47.0	50.8	81.2	77.6
4-H-3-M-B	4-OH	85.2	83.7	79.8	184.4	118.9	110.8	214.5	11.3	19.8	336.7	39.6	41.3	63.7	90.6	89.4
4-H-3-M-C	4-OH	81.6	79.5	76.8	174.6	112.2	104.4	222.6	13.7	19.5	331.7	39.7	41.8	65	86.3	82.1
4-H-3-M-P	4-OH	82.8	79.6	76.8	177.2	112.6	103.5	219.4	13.7	20.5	347.1	45.5	47.0	50.9	80.6	77.0
4-H-3,5-DM-B	4-OH	80.8	78.9	77.3	180.3	115.8	108.3	218.7	9.7	16.1	372	39.4	41.7	58.5	86.0	82.7
4-H-3,5-DM-C	4-OH	78.0	75.7	71.3	168.8	110.2	102.7	220.9	12.0	17.5	294.4	39.2	41.0	61.1	83.0	79.2
4-H-3,5-DM-P	4-OH	78.9	75.5	72.8	166.2	108.3	100.9	226.5	13.5	19.0	348.6	45.6	46.6	45.6	76.4	73.3

The introduction of the methoxy group and phenolic hydroxyl group also reduce the BDE of the phenolic hydroxyl group of phenolic acids, which corresponds to higher antioxidant activities in experimental results (Fig. 1) and in particular, 4-OH BDE of 4-H-3,5-DM-B in ethanol, which is 10 kcal/mol lower than 4-OH BDE of 4-H-B. Compared with 3-H-B and 4-H-B in ethanol, the 3-OH and 4-OH BDEs of 3,4-DH-B decrease to 4.1 kcal/mol and 5.6 kcal/mol, respectively. Moreover, the OH BDE of 4-H-3-M-C and 4-OH BDE of 3,4-DH-C are 4.3 kcal/mol and 6.5 kcal/mol lower than that of 4-H-C, respectively, which is close to the 2.3 kcal/mol and 6.6 kcal/mol calculated by Chen *et al*.^18^.

However, in the DPPH system, the order of RSA values of 4-H-3,5-DM is B ≈ C < P (Fig. 1 and Table S1), whereas the order of the BDE values of 4-H-3,5-DM in the ethanol phase are B (78.9 kcal/mol) > C (75.7 kcal/mol) ≈ P (75.5 kcal/mol) (Table 2). The two results obviously do not correspond with each other. Therefore, we calculate the ratio of the phenolic hydroxyl group in the active site of phenolic acid to the amount of DPPH^•^ in the DPPH system. The calculation process and formula of the ratio are shown in the Supplementary Data (Table S2). Their ratios are P (2.1) > C (1.6) ≈ B (1.5) (Table S2), which indicates that each -OH of 4-H-3,5-DM-P/C/B scavenged about 2.1, 1.6, and 1.5 DPPH^•^, respectively. When each phenolic hydroxyl scavenged more than one DPPH^•^ in the proton solvent ethanol reaction system, quinones may have a nucleophilic reaction with ethanol, resulting in the regeneration of the phenolic hydroxyl structure^24^. Alternatively, after scavenging DPPH^•^, a few semiquinones formed by ferulic acid may couple to form dimers, which increases the scavenging activity of the free radicals^25^. In fact, the formation of a sinapic acid dimer is detected in the DPPH reaction system using HPLC-MS (Fig. S1). Therefore, the final result is B ≈ C < P due to the adverse effects of the possible intramolecular hydrogen bond mentioned above. Moreover, the BDE of 4-H-C (83.8 kcal/mol) is not significantly different from that of 4-H-P (83.9 kcal/mol) in ethanol, but the DPPH^•^ scavenging activity of the former is higher than that of the latter, which is consistent with the DPPH^•^ ratio of 4-H-C > 4-H-P (Table S2). These results imply that the effects of thermodynamics and kinetics should be considered together when the amount of hydroxyl phenol is greater than that of DPPH^•^ and the BDE value is too high. Previous studies also have shown that BDE can only roughly evaluate the antioxidant activity under polar solvent conditions^26^. As a result, in the DPPH reaction system, HAT may not be the main mechanism.

#### SET-PT mechanism

The IP and PDE values are the reaction enthalpies related to the SET-PT mechanism. The PDE value is related to the PA of the phenolic acid cation free radicals. The IP and PDE values are greatly influenced by solvent polarity^27^. As shown in Table 2, while the order of the PDE value is gas > water > ethanol, the PDE value of the protons in gas is generally higher than in ethanol. The proton dissociation ability of the molecule in ethanol is stronger than that in water, which is mainly influenced by the enthalpy of proton solvation. It was reported that the difference of PDE values between *p*-phenylenediamine in the gas phase and in methanol was more than 200 kcal/mol^28^, which was similar to the results in this study. The solvation enthalpy of protons in the ethanol system was −249.8 kcal/mol, whereas that of protons in the water system was −244.3 kcal/mol, which were also consistent with our calculations^27^.

The IP value is influenced by the overall structure of the molecule and the delocalization and conjugation of the pion electrons, which can directly reflect the electron donation ability of the molecule^23^. The lower the IP value, the easier it is for the molecule to donate electrons. In this study, for the same molecule, the order of the IP value is gas > ethanol > water, indicating that the stronger the solvent polarity, the higher the electron-donating ability of the molecule. Compared with -COOH in the three micro-environments, -CH_2_COOH and -CH = CHCOOH reduce the IP value of the phenolic acids by 8.2 kcal/mol and 7.8 kcal/mol on average, respectively, which indicates that -CH_2_COOH and -CH = CHCOOH significantly enhances the electron donation ability. The introduction of the methoxy and hydroxyl groups also reduce the IP value. For example, the IP values of 4-H-P are 20.6 kcal/mol higher than 4-H-3,5-DM-P and 6.6 kcal/mol higher than 3,4-DH-P (Table 2). It was reported that the IP value of dihydrochalcone with four phenolic hydroxyl substituents was lower than that of dihydrochalcone with three phenolic hydroxyl substituents^29^. In fact, a similar principle can be obtained with hydroxyphenylacetic and hydroxycyancinnamic acids. The lower the IP value of phenolic acid, the higher is the antioxidant activity, which corresponds to the previous experimental results.

**Figure 2:**
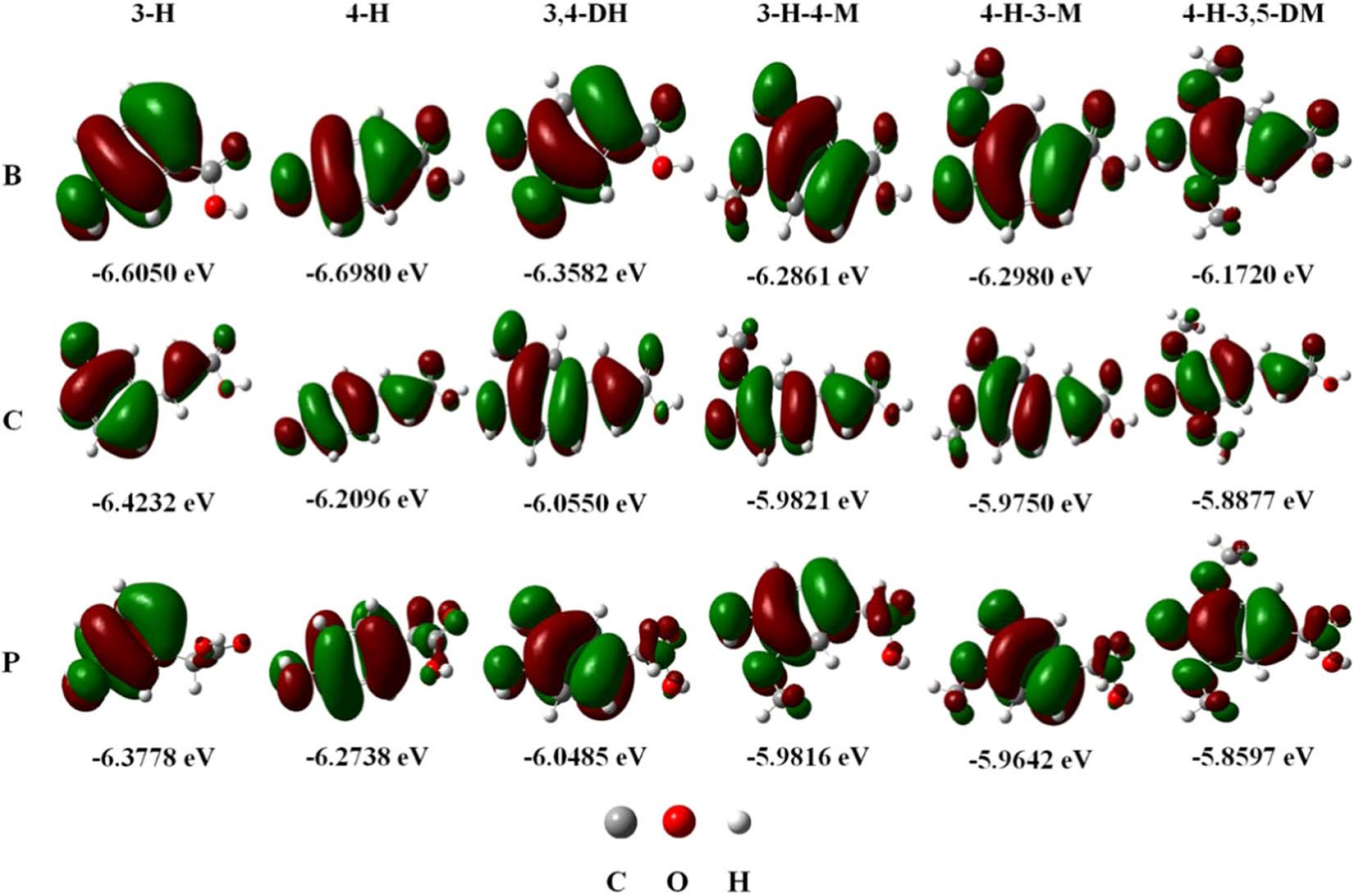
The energy and distribution of HOMO for18 tested compounds in ethanol.

Generally speaking, as with the IP, the energy of the frontier orbitals is also an important parameter that reflects the electron-donating ability of a molecule. Larger values of HOMO indicate that the electron-donating ability is stronger^29,30^. We use the B3LYP/6-311++G (d, p) and M06-2X /6–311++G (d, p) methods to calculate the HOMO energy of 18 phenolic acids in the gas phase, water, and ethanol, respectively (Table S3). Taking the calculation results in ethanol as an example, horizontally (Fig. 2), the HOMO value gradually increases from left to right, in particular, the single hydroxyl structure (3-H and 4-H) has a large difference compared with other molecules, which are basically consistent with the results of the DPPH and FRAP assays. However, it is not appropriate to make a strict horizontal comparison between the computational results and the experimental results, because the HOMO energy is calculated on the basis of -OH, the number and the position of -OH in each molecule are very important. Vertically (Fig. 2), the position and number of -OH in each molecule are the same. The order of HOMO energy gradually increases from the top to the bottom (P > C > B) with the exception of 4-H, showing that C > P > B, which almost corresponds to the results of the DPPH and FRAP assays. Interestingly, the principle of HOMO energy in gas is the same as that in ethanol (Table S3), which indicates that the antioxidant activity of phenolic acids can also be explained by HOMO values in gas and ethanol. However, the order of HOMO energy in water is P > C > B, except with 3-H and 4-H-3,5-DM (Table S3), therefore the solvent effect has a certain impact on the HOMO energy value of the molecule. In addition, the HOMO energy of each molecule using the M06-2X method is more than 1 eV smaller than that of the B3LYP. In gas, the trends of the two results are the same (C > P > B), including the order of HOMO energy in 4-H. However, in ethanol and water, the order of all molecules is C > P > B. This shows that the method and micro-environment also have an effect on the HOMO values of phenolic acids. On the whole, the HOMO values (Fig. 2 and Table S3), IP values (Table 1), and experimental data (Fig. 1) of hydroxybenzoic acid are significantly lower than those of hydroxyphenylacetic acid (P > C) and hydroxycinnamic acid (B > C), respectively. While these differences between phenylacetic acid and cinnamic acid are small, their SAR can be easily affected by the reaction system, solvent effect, and calculation method, with the results floating up and down.

#### SPLET mechanism

The PA value and ETE value are the enthalpy of the reaction related to the SPLET mechanism. The PA value represents the degree of difficulty of phenolic hydroxyl dephosphorization, while the ETE value represents the electron donation ability of corresponding polyphenol ions. As shown in Table 2, similar to the PDE values, molecule dephosphorization in ethanol was easier than in water. Due to the direct influence of proton solvation enthalpy, the order of PA values is gas > water > ethanol, whereas due to the influence of electron solvation enthalpy, the order of ETE values is ethanol > water > gas. The differences between the average PA of phenolic acids in ethanol and water and the average PA in gas are 293.7 kcal/mol and 291.6 kcal/mol, respectively, which are close to the reported average PA values of polyphenols in methanol and water, 287.5 kcal/mol and 281.5 kcal/mol, respectively^29^. Compared with the IP values, the ETE values are generally smaller, indicating that electrons are donated easier by polyphenol anions than by neutral molecules, for example, catechol anions have stronger electron-donating ability than neutral molecules^31^.

In this study, compared with -COOH, -CH_2_COOH reduces the ETE value of phenolic acids by 8.1 kcal/mol on average, while -CH = CHCOOH reduces the ETE value of phenolic acid molecules by 1.6 kcal/mol on average. Therefore, the TEAC difference in the reduction ability of Fe(III) between hydroxyphenylacetic acid and hydroxybenzoic acid is significantly greater than that between hydroxycinnamic acid and hydroxybenzoic acid in the FRAP assay (Fig. 1B). Moreover, taking 4-H-3, 5-DM-B, and 4-H-B as examples, the differences in the ETE value between them in gas, ethanol, and water are more than 10 kcal/mol, respectively (Table 2), indicating that the methoxy group decreases the ETE value of the phenolic hydroxyl group.

In fact, the electron donor group increases the acidity of the phenolic hydroxyl group and decreases the pKa value, which is more conducive to proton dissociation and the antioxidant activity. However, the effect of pH on proton dissociation ability is not considered in this calculation. Nevertheless, since the FRAP assay is tested in water with pH 3.6, the consistency between the ETE values and the TEACs of FRAP is greater than the RSA of the DPPH assay. Therefore, we speculate that SPLET is the main reaction mechanism in the FRAP system.

The calculation results show that the BDE values of the HAT mechanism in gas are the lowest relative to the IP values of the SET-PT mechanism and the PA values of SPLET mechanism, so HAT is the most likely mechanism to occur in gas. The PA values in water and ethanol are significantly lower than the IP value, so the SPLET mechanism is prone to occur in the two environments. Moreover, except for monohydroxyphenolic acid, the ETE values of phenolic acids are consistent with the experimental results of the FRAP assay. SPLET may be the main reaction mechanism in the FRAP system. In the DPPH system, although SPLET has thermodynamic advantages, some experimental results can be explained by the HAT and SET-PT mechanisms. Therefore, all three mechanisms may occur in the DPPH system.

## Conclusion

The experimental results show that -CH_2_COOH and -CH = CHCOOH promote the antioxidant activities of phenolic acids when the other substituents on the benzene ring are the same, which may be related to the electron donation ability of the functional groups. The introduction of methoxy and phenolic hydroxyl groups can promote the antioxidant activities of phenolic acids. On the other hand, compared with -COOH, the BDE, PA, and ETE of the phenolic hydroxyl group can be reduced due to the introduction of -CH_2_COOH and -CH = CHCOOH. A methoxy group can reduce the BDE of the phenolic hydroxyl group and enhance the electron-donating ability of phenolic acids by reducing PA and ETE values. Therefore, by comparing the results of the experiment and calculation, we speculate that HAT, SET-PT, and SPLET mechanisms may occur in DPPH reaction system, and different molecules are affected by reaction thermodynamics. Whereas SPLET is considered the main reaction mechanism in the FRAP system because ETE values are consistent with the experiment results, except with the 4-H group. These results may help us to evaluate the reaction mechanism of phenolic acids and screen them for pharmaceutical and food applications based on their structure.

## Methods

### Chemicals

All 18 phenolic acids were of chromatographic-reagent grade purchased from Aladdin Industrial Inc. (Shanghai, China). Other chemicals and reagents were of analytical-reagent grade and purchased from Sigma Chemical Co (China).

### DPPH assay

The DPPH·, the stable artificial free radicals, has been widely used for the measurement of free radical scavenging capacity of the phenolic compounds in ethanol and aqueous systems^32^. Briefly, 2 mL DPPH solution (0.2 mM, in 95% ethanol) was incubated with 2 mL different concentrations of phenolic acid solution. Then, the reaction mixture was shaken and incubated in the dark for 40 min at room temperature. The absorbance was immediately recorded at 517 nm against ethanol with a spectrophotometer (Metash, model UV-5200, China). The DPPH free radical scavenging rate was calculated using the equation:

$${\rm{DPPH}}\,{\rm{scavenging}}\,{\rm{activity}}\,( \% )=\frac{{{\rm{A}}}_{0}-{{\rm{A}}}_{1}}{{{\rm{A}}}_{0}}\times 100.$$

where A_0_ was the absorbance of the control reaction (containing all reagents except the tested compound), and A_1_ was the absorbance of the test reaction (containing all reagents with the tested compound). The percentage of DPPH radical scavenging activity was plotted against the sample concentration to acquire the IC_50_ value, defined as the concentration of sample necessary to cause 50% inhibition. Radical Scavenging Activity (RSA) was calculated from the IC_50_ value as the equation: RSA = pIC_50_ = −lg[IC_50_]. The smaller the IC_50_ value, the larger is the RSA value and the higher is the antioxidant activity.

### FRAP assay

The FRAP assay was used to determine the AC of phenolic acids by the reduction of Fe(III) and Fe (II)^9^. Briefly, Fe(III) was reduced to Fe(II), and Fe(II) was mixed with TPTZ to form a blue complex with strong absorption peak at 593 nm at pH = 3.6 condition. Acetate buffer (pH = 3.6), TPTZ solution (10 mM, in 40 mM hydrochloric acid) and FeCl_3_ solution (20 mM, in water) were mixed in a ratio of 10:1:1 to prepare FRAP working solution. Phenolic acid solution (0.5 mL) was mixed with 4.0 mL FRAP working solution, and reacted at 37 °C for 30 min in the dark, and the absorbance at 593 nm was immediately recorded with a spectrophotometer. The result was expressed as the equivalent amount of Trolox per mole of phenolic acid (mol TE/mol).

### Computational methods

Here, the BDE, IP, PDE, PA and ETE values were determined in the gas phase, water and ethanol at 298.15 K and 1 atmosphere based on the following expressions (Eqs. 2, 5, 6, 9 and 10)^33,34^, respectively. The enthalpy values of hydrogen atom in the gas phase was -312.956 kcal/mol^29^ and in solvents was from Parker *et al*.^35^. The enthalpy values of protons and electrons in the gas phase and solvents were from Rimarcik *et al*.^28^.

Hydrogen-Atom Transfer (HAT) mechanism

1 $${{\rm{R}}}^{\bullet }+{\rm{ArOH}}\to {\rm{RH}}+{{\rm{ArO}}}^{\bullet }$$

2 $${\rm{BDE}}={\rm{H}}({{\rm{ArO}}}^{\bullet })+{\rm{H}}({{\rm{H}}}^{\bullet })-{\rm{H}}({\rm{ArOH}})$$

Single electron transfer followed by proton transfer (SET-PT) mechanism

3 $${{\rm{R}}}^{\bullet }+{\rm{ArOH}}\to {{\rm{R}}}^{-}+{{\rm{ArOH}}}^{\bullet +}$$

4 $${{\rm{ArOH}}}^{\bullet }+\to {{\rm{ArO}}}^{\bullet }+{{\rm{H}}}^{+}$$

5 $${\rm{IP}}={\rm{H}}({{\rm{ArOH}}}^{\bullet +})+{\rm{H}}({{\rm{e}}}^{-})-{\rm{H}}({\rm{ArOH}})$$

6 $${\rm{PDE}}={\rm{H}}({{\rm{ArO}}}^{\bullet })+{\rm{H}}({\rm{H}}+)-({{\rm{ArOH}}}^{\bullet +})$$

Sequential proton-loss electron transfer (SPLET) mechanism

7 $${\rm{ArOH}}\to {{\rm{ArO}}}^{-}+{{\rm{H}}}^{+}$$

8 $${\rm{ETE}}={\rm{H}}({{\rm{ArO}}}^{\bullet })+{\rm{H}}({{\rm{e}}}^{-})-{\rm{H}}({{\rm{ArO}}}^{-})$$

9 $${{\rm{ArO}}}^{-}+{{\rm{R}}}^{\bullet }+{{\rm{H}}}^{+}\to {\rm{RH}}+{{\rm{ArO}}}^{\bullet }$$

10 $${\rm{PA}}={\rm{H}}({{\rm{ArO}}}^{-})+{\rm{H}}({{\rm{H}}}^{+})-{\rm{H}}({\rm{ArOH}})$$

All the calculations were carried out using the Gaussian 09 program suite. The geometries were obtained using the B3LYP/6-311++G (d, p) and UB3LYP were used to optimize free radical system^36^. Here, the wave functions of every radical were checked after calculation, and all spin contaminations of radicals were controlled to avoid affecting the calculation of energy value^18^. The Cartesian coordinates of each molecules used in this study were shown in the Supplementary Data (Figs. S2–S19). The absence of imaginary frequencies confirmed that the optimized structure was a local minimum^37^. The B3LYP/6-311++G (d, p) and M06-2 X /6-311++G (d, p) methods were also used to calculate the HOMO energies of molecules. The solvent effects were computed using an integral equation formalism polarized continuum model (IEF-PCM method)^38,39^.

### Statistical analysis

All determinations in Fig. 1 represented the means of at least three independent experiments, each conducted in triplicate. The data were expressed as mean ± standard deviation (SD) and assessed with one-way analysis of variance (ANOVA) using SPSS 19.0 software (IBM, New York, USA). Significant differences between means were determined using Duncan’s multiple tests (*p* < 0.05).

## Supplementary information

Dataset1.
