# Author Correction: Structure-antioxidant activity relationship of methoxy, phenolic hydroxyl, and carboxylic acid groups of phenolic acids

**DOI:** 10.1038/s41598-020-62493-y

**Published:** 2020-03-24

**Authors:** Jinxiang Chen, Jing Yang, Lanlan Ma, Jun Li, Nasir Shahzad, Chan Kyung Kim

**Affiliations:** 1 School of Chemical Engineering and Technology, North University of China, Taiyuan, 030051 China; 2 Department of Chemistry and Chemical Engineering, Inha University, Incheon, 22212 Korea

Correction to: *Scientific Reports* 10.1038/s41598-020-59451-z, published online 13 February 2020

This Article contains typographical errors in Equation (4), where

$$ArO{H}^{\bullet }+\to Ar{O}^{\bullet }+{H}^{+}$$

should read:

$$ArO{H}^{\bullet +}\to Ar{O}^{\bullet }+{H}^{+}$$

In addition, equation (6)

$$PDE=H(Ar{O}^{\bullet })+H(H+)-(ArO{H}^{\bullet +})$$

should read:

$$PDE=H(Ar{O}^{\bullet })+H({H}^{+})-H(ArO{H}^{\bullet +})$$

