# Supplementary material for: Structure-antioxidant activity relationship of methoxy, phenolic hydroxyl, and carboxylic acid groups of phenolic acids
Source: Sci Rep. 2020 Feb 13;10:2611. doi: 10.1038/s41598-020-59451-z (PMC7018807; doi:10.1038/s41598-020-59451-z)
Supplement: Supplementary file 1 — Dataset1. [file 41598_2020_59451_MOESM1_ESM.docx]

**Structure-antioxidant activity relationship of methoxy, phenolic hydroxyl, and carboxylic acid groups of phenolic acids**

Jinxiang Chen^1^, Jing Yang^1*^, Lanlan Ma^1^, Jun Li^1^, Nasir Shahzad^2^, Chan Kyung Kim^2*^

^1^ School of Chemical Engineering and Technology, North University of China, Taiyuan 030051, China

^2^ Department of Chemistry and Chemical Engineering, Inha University, Incheon 22212, Korea

Correspondence and requests for materials should be addressed to J. Y (email:[yangjing5152@163.com](mailto:yangjing5152@163.com)) or C. K. K. (email: [kckyung@inha.ac.kr)](mailto:kckyung@inha.ac.kr))

| **Compounds** | **RSA_DPPH_** | | |
| --- | --- | --- | --- |
|  | **Hydroxybenzoic acid** | **Hydroxycinnamic acid** | **Hydroxyphenylacetic acid** |
| 3-H | < 0 | 0.835 ± 0.035Eβ | 0.979 ± 0.038Cα |
| 4-H | < 0 | 1.211 ± 0.080Dα | 0.911 ± 0.071Cβ |
| 3,4-DH | 4.287 ± 0.030Aγ | 4.544 ± 0.040Aβ | 4.847 ± 0.029Aα |
| 3-H-4-M | 2.471 ± 0.040Bγ | 2.799 ± 0.030Cβ | 4.160 ± 0.108Bα |
| 4-H-3-M | 2.423 ± 0.061Bγ | 3.570 ± 0.056Bβ | 4.156 ± 0.123Bα |
| 4-H-3,5-DM | 4.363 ± 0.061Aβ | 4.378 ± 0.015Aβ | 4.729 ± 0.029Aα |
|  | **TEAC_FRAP_** | | |
| 3-H | 0.003 ± 0.001Dβ | 0.005 ± 0.001Eβ | 0.007 ± 0.001Dα |
| 4-H | 0.002 ± 0.001Dβ | 0.010 ± 0.001Dα | 0.005 ± 0.001Dβ |
| 3,4-DH | 1.467 ± 0.056Aγ | 1.945 ± 0.035Aβ | 3.209 ± 0.112Aα |
| 3-H-4-M | 0.155 ± 0.010Cγ | 0.327 ± 0.025Cβ | 0.597 ± 0.009Cα |
| 4-H-3-M | 0.165 ± 0.019Cγ | 0.329 ± 0.023Cβ | 0.652 ± 0.030Cα |
| 4-H-3,5-DM | 1.128 ± 0.037Bγ | 1.271 ± 0.031Bβ | 2.508 ± 0.048Bα |

**Table S1.** RSA values of DPPH and TEAC value of FRAP in 18 tested compounds. The data were expressed as mean (±SD) (n=3). Different lowercase Greek letters represent different phenolic acids with the same methoxy and phenolic hydroxyl groups (*p* < 0.05), and different Latin letters represented different phenolic acids with the same carboxylic acid group (*p* < 0.05).

| **Compounds** | **IC_50_ (mM)** | **Ratios** |
| --- | --- | --- |
| 3-H-B | > 1000 | < 0.001 |
| 3-H-C | 146.367 ± 2.555 | < 0.001 |
| 3-H-P | 104.907 ± 1.005 | < 0.001 |
| 4-H-B | > 1000 | < 0.001 |
| 4-H-C | 61.503 ± 0.555 | 0.001 ± 0.000 |
| 4-H-P | 122.728 ± 1.635 | < 0.001 |
| 3,4-DH-B | 0.052 ± 0.002 | 0.885 ± 0.046 |
| 3,4-DH-C | 0.029 ± 0.001 | 1.167 ± 0.028 |
| 3,4-DH-P | 0.014 ± 0.001 | 2.346 ± 0.224 |
| 3-H-4-M-B | 3.777 ± 0.160 | 0.018 ± 0.001 |
| 3-H-4-M-C | 1.587 ± 0.006 | 0.042 ± 0.001 |
| 3-H-4-M-P | 0.091 ± 0.007 | 0.731 ± 0.056 |
| 4-H-3-M-B | 3.383 ± 0.053 | 0.020 ± 0.001 |
| 4-H-3-M-C | 0.269 ± 0.005 | 1.243 ± 0.123 |
| 4-H-3-M-P | 0.074 ± 0.004 | 0.898 ± 0.049 |
| 4-H-3,5-DM-B | 0.043 ± 0.002 | 1.537 ± 0.073 |
| 4-H-3,5-DM-C | 0.042 ± 0.001 | 1.593 ± 0.034 |
| 4-H-3,5-DM-P | 0.021 ± 0.002 | 2.145 ± 0.104 |
| Toxlox | 0.031 ± 0.002 | 2.149 ± 0.098 |

**Table S2.** The ratio of DPPH• to phenolic hydroxyl in phenolic acids at IC_50_ concentration.

$$R\text{atio}=\frac{C\times\text{V}_{1}\times0.5}{IC_{50}\times\text{n}\times\text{V}_{2}}$$

C: The concentration of DPPH• solution (mM);

V_1_: Volume (mL) of DPPH• in the reaction system;

n: The number of phenolic hydroxyl groups in each molecule;

V_2_: The total volume of the reaction system (mL).

| **Compounds** | **B3LYP-HOMO (eV)** | | | **M062X-HOMO (eV)** | | |
| --- | --- | --- | --- | --- | --- | --- |
|  | **gas** | **water** | **ethanol** | **gas** | **water** | **ethanol** |
| 3-H-B | -6.7709 | -6.6368 | -6.6050 | -8.3638 | -8.0212 | -7.9850 |
| 3-H-C | -6.6395 | -6.5244 | -6.4232 | -7.9197 | -7.7731 | -7.7295 |
| 3-H-P | -6.4964 | -6.4031 | -6.3778 | -7.6817 | -7.7965 | -7.7666 |
| 4-H-B | -6.8561 | -6.7318 | -6.6980 | -8.4424 | -8.0776 | -8.0397 |
| 4-H-C | -6.4088 | -6.2363 | -6.2096 | -7.6359 | -7.5004 | -7.4708 |
| 4-H-P | -6.4137 | -6.3141 | -6.2738 | -7.7374 | -7.6781 | -7.6531 |
| 3,4-DH-B | -6.5046 | -6.4006 | -6.3582 | -7.8169 | -7.7513 | -7.7048 |
| 3,4-DH-C | -6.2858 | -6.0874 | -6.0550 | -7.5255 | -7.3663 | -7.3315 |
| 3,4-DH-P | -6.1911 | -6.0920 | -6.0485 | -7.5217 | -7.4765 | -7.4300 |
| 3-H-4-M-B | -6.3690 | -6.3301 | -6.2861 | -8.1056 | -7.6915 | -7.6444 |
| 3-H-4-M-C | -6.1519 | -6.0148 | -5.9821 | -7.4014 | -7.3067 | -7.2711 |
| 3-H-4-M-P | -6.0501 | -6.0281 | -5.9816 | -7.3962 | -7.3788 | -7.3722 |
| 4-H-3-M-B | -6.4063 | -6.4090 | -6.2980 | -8.1274 | -7.6963 | -7.6539 |
| 4-H-3-M-C | -6.1182 | -6.0093 | -5.9750 | -7.3432 | -7.2890 | -7.2501 |
| 4-H-3-M-P | -6.0180 | -6.0240 | -5.9642 | -7.3413 | -7.4028 | -7.3543 |
| 4-H-3,5-DM-B | -6.1225 | -6.2338 | -6.1720 | -7.4324 | -7.5978 | -7.5304 |
| 4-H-3,5-DM-C | -5.9301 | -5.9367 | -5.8877 | -7.1598 | -7.2234 | -7.1693 |
| 4-H-3,5-DM-P | -5.7598 | -5.9138 | -5.8597 | -7.0868 | -7.3094 | -7.2411 |

Table S3. The HOMO energy of 18 phenolic acids calculated by the B3LYP and M062X methods.

B

A

Figure S1. Chromatogram (A) and mass spectrum (B) of sinapic acid dimer in the DPPH system.

***HPLC-MS analysis.*** HPLC-MS analyses were performed on a Thermo Scientific™ Exactive plus/Q Exactive HPLC-MS system (Thermo Fisher Scientific Inc, USA). Samples were performed with a Thermo Hypersil GOLD C18 column (100 mm × 2.1 mm, 3-μm particle size, Thermo Fisher Scientific Inc). A gradient program was divided (phase A water + 0.1% formic acid) and phase B (acetonitrile). The flow rate was 0.3 mL/min, and the phase gradient was as follows:10% B at 0–1.5 min; 10%–25% B at 1.5–8 min; 25%–70% at 8–9 min; 70%–10% at 9–10.5 min; and 10% B at 10.5–12 min. The effluents were directed into the MS via the electrospray interface. Nitrogen was used as the nebulizing and drying gas. The detector (mass spectrum) operated under the following conditions: electrospray ionization (ESI) interface operating in negative mode; source: 3.5 kV; sheath gas flow rate:40, auxiliary gas flow:15; capillary temperature:320 °C and heater temperature:300 °C. HPLC-MS was recorded over a mass-to-charge (*m/z*) range of 100 to 1,000.


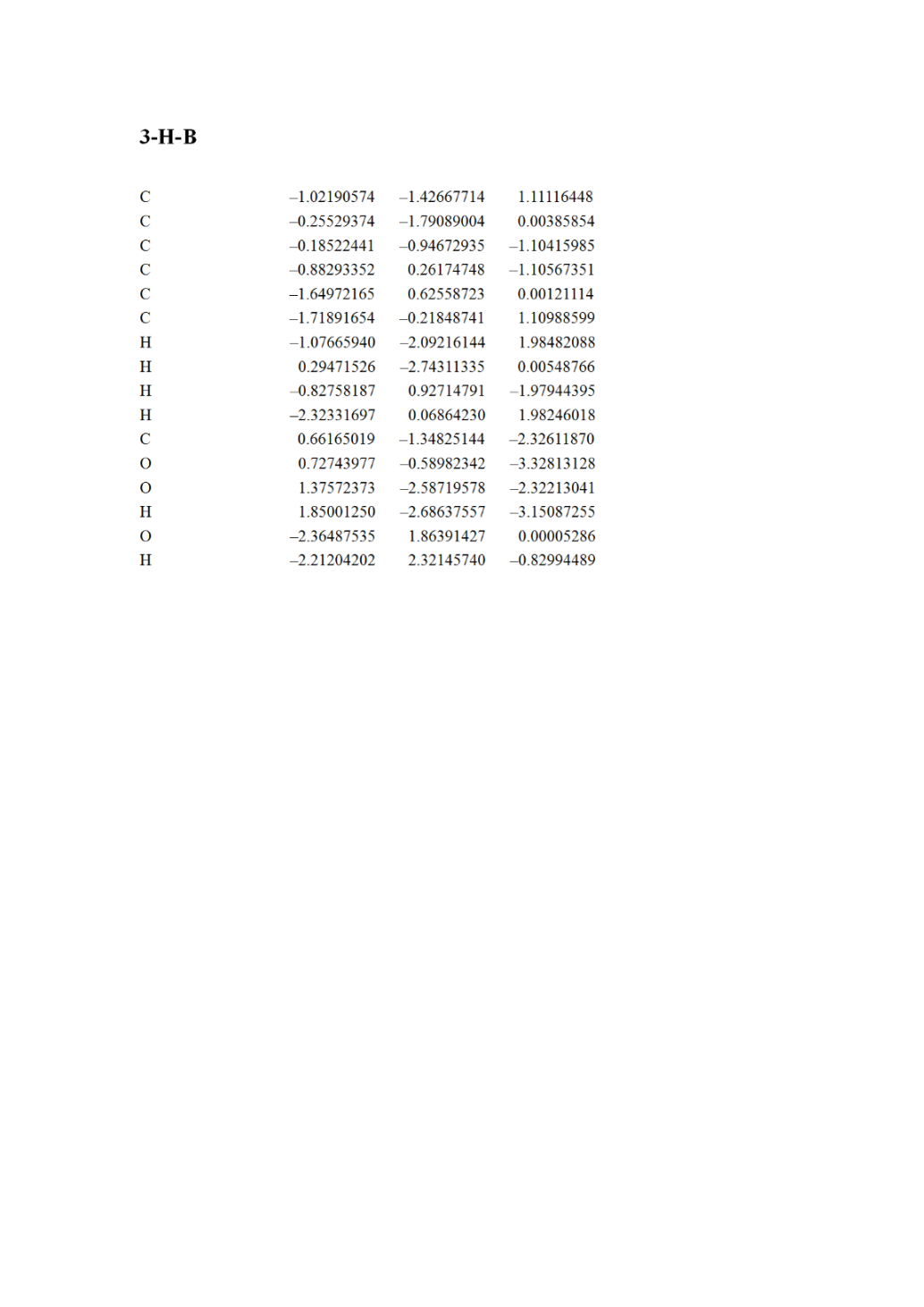


Figure S2. The Cartesian coordinates for 3-H-B used in this study.


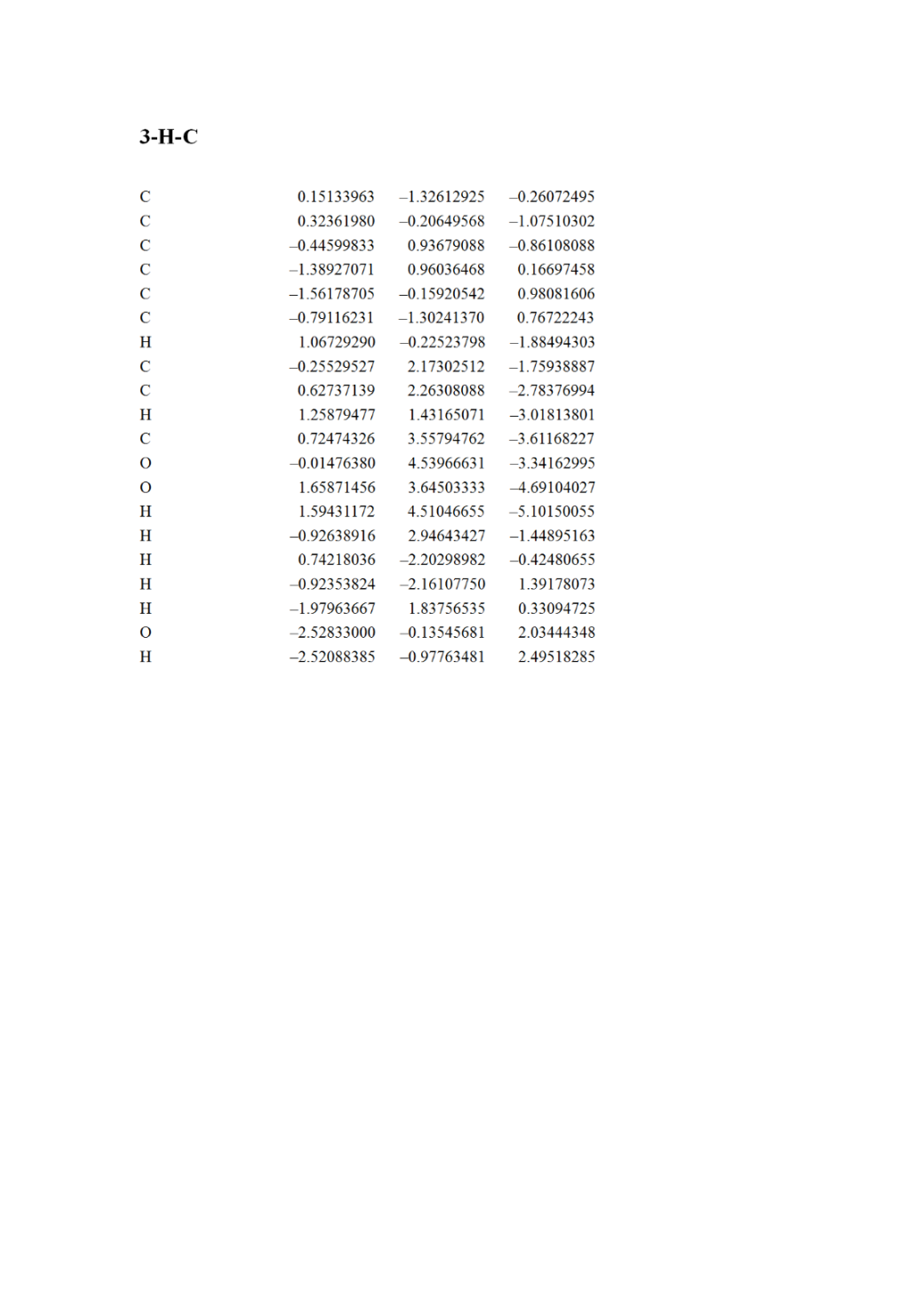


Figure S3. The Cartesian coordinates for 3-H-C used in this study.


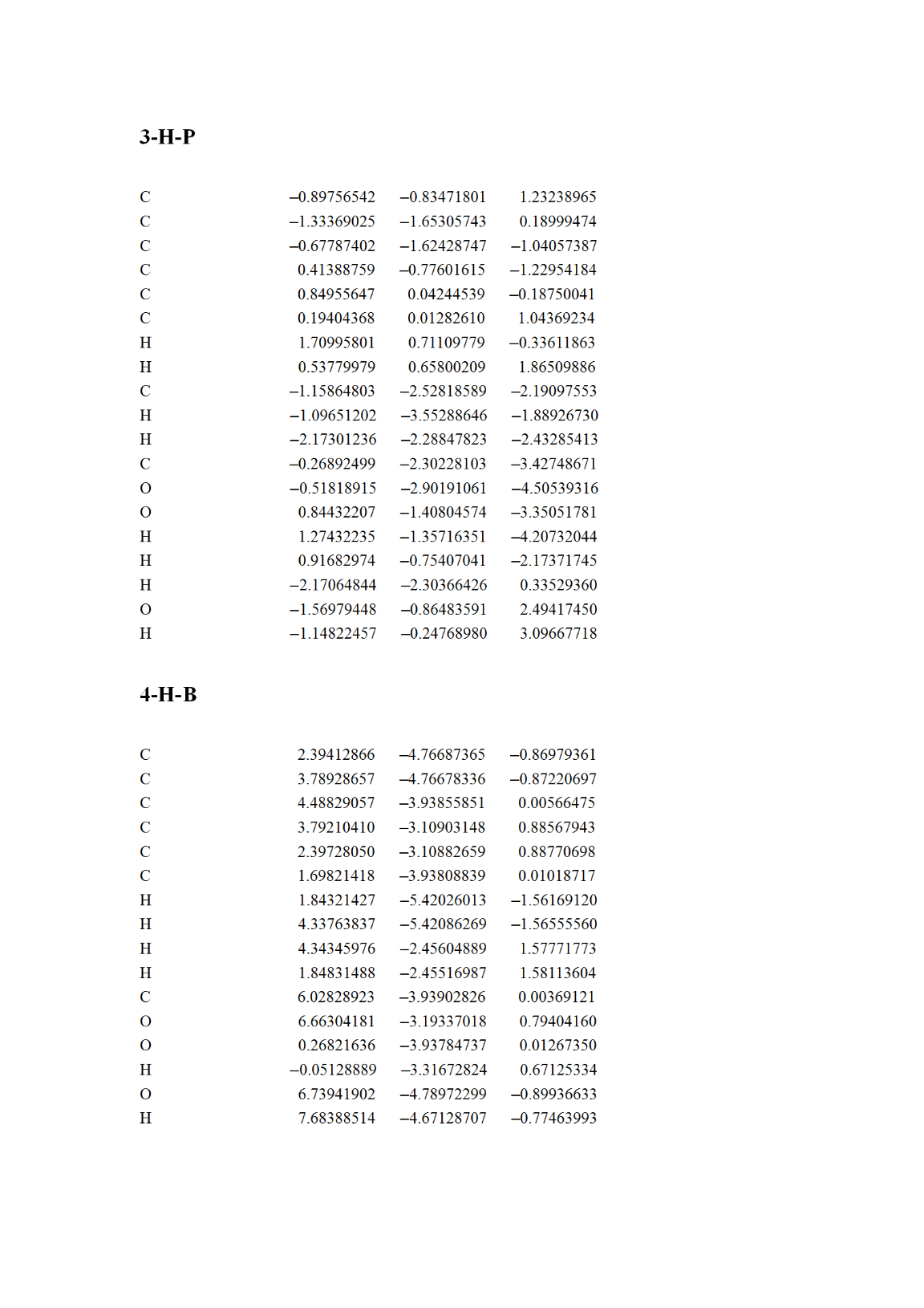


Figure S4. The Cartesian coordinates for 3-H-P used in this study.


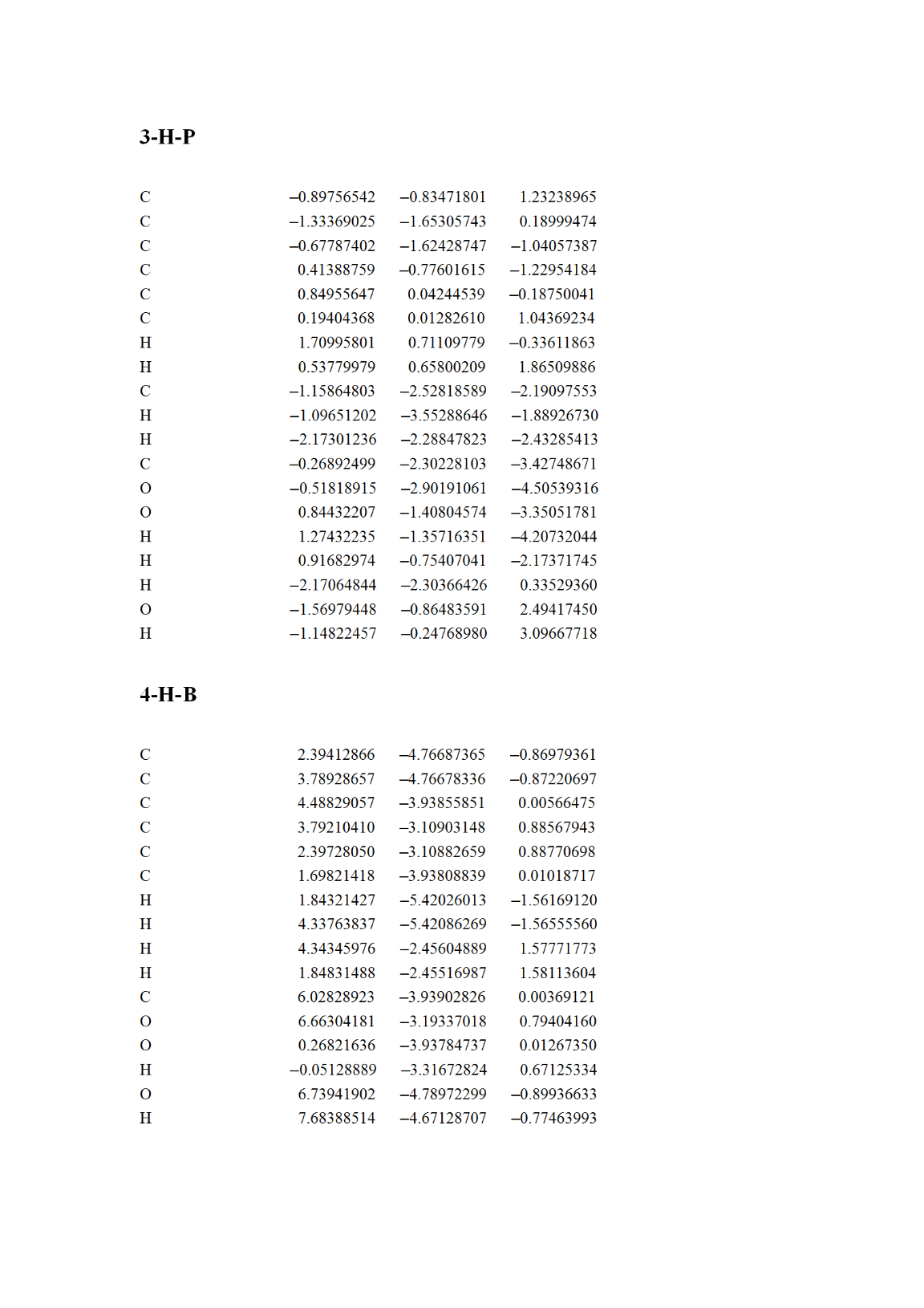


Figure S5. The Cartesian coordinates for 4-H-B used in this study.


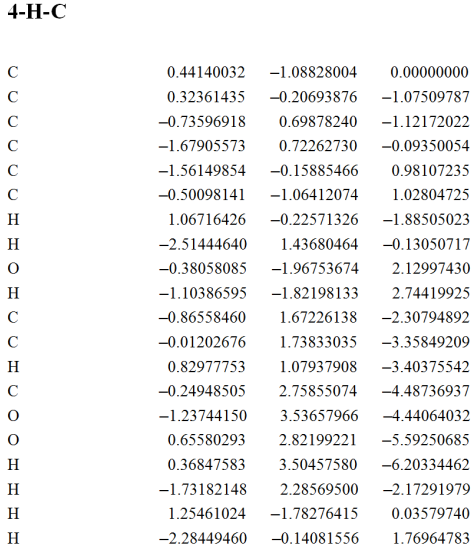


Figure S6. The Cartesian coordinates for 4-H-C used in this study.


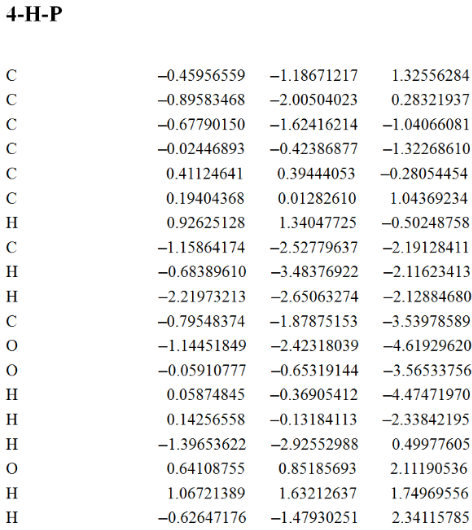


Figure S7. The Cartesian coordinates for 4-H-P used in this study.


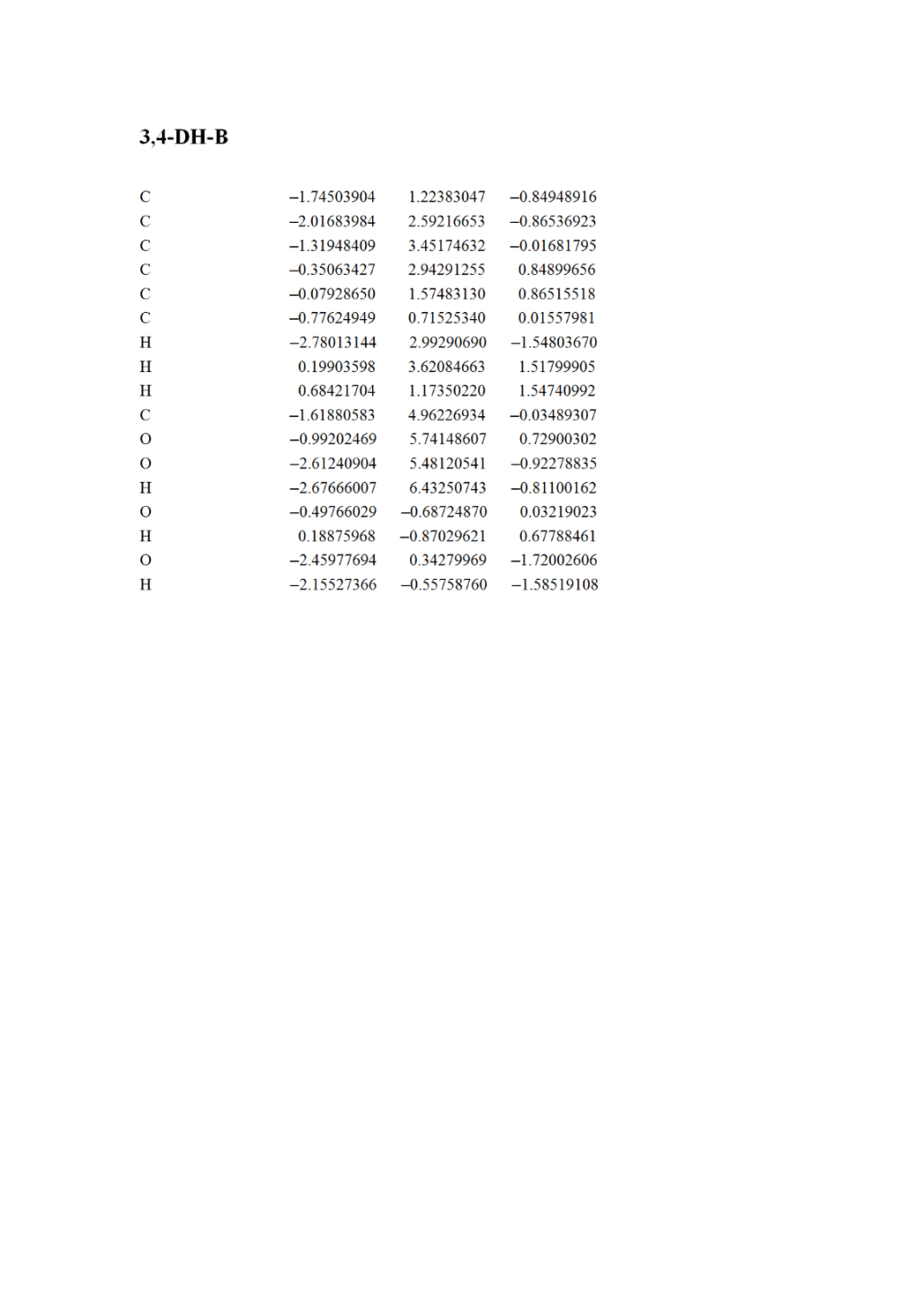


Figure S8. The Cartesian coordinates for 3,4-DH-B used in this study.


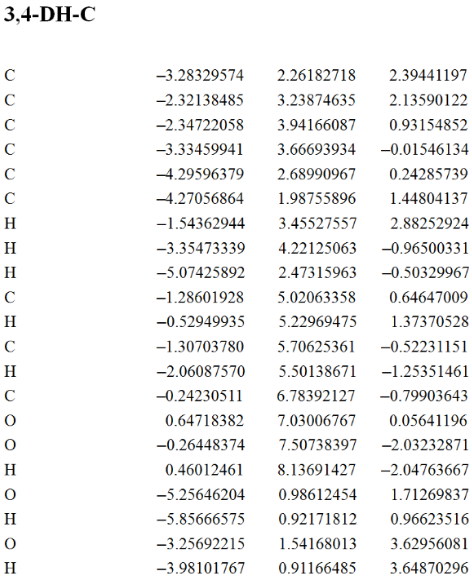


Figure S9. The Cartesian coordinates for 3,4-DH-C used in this study.


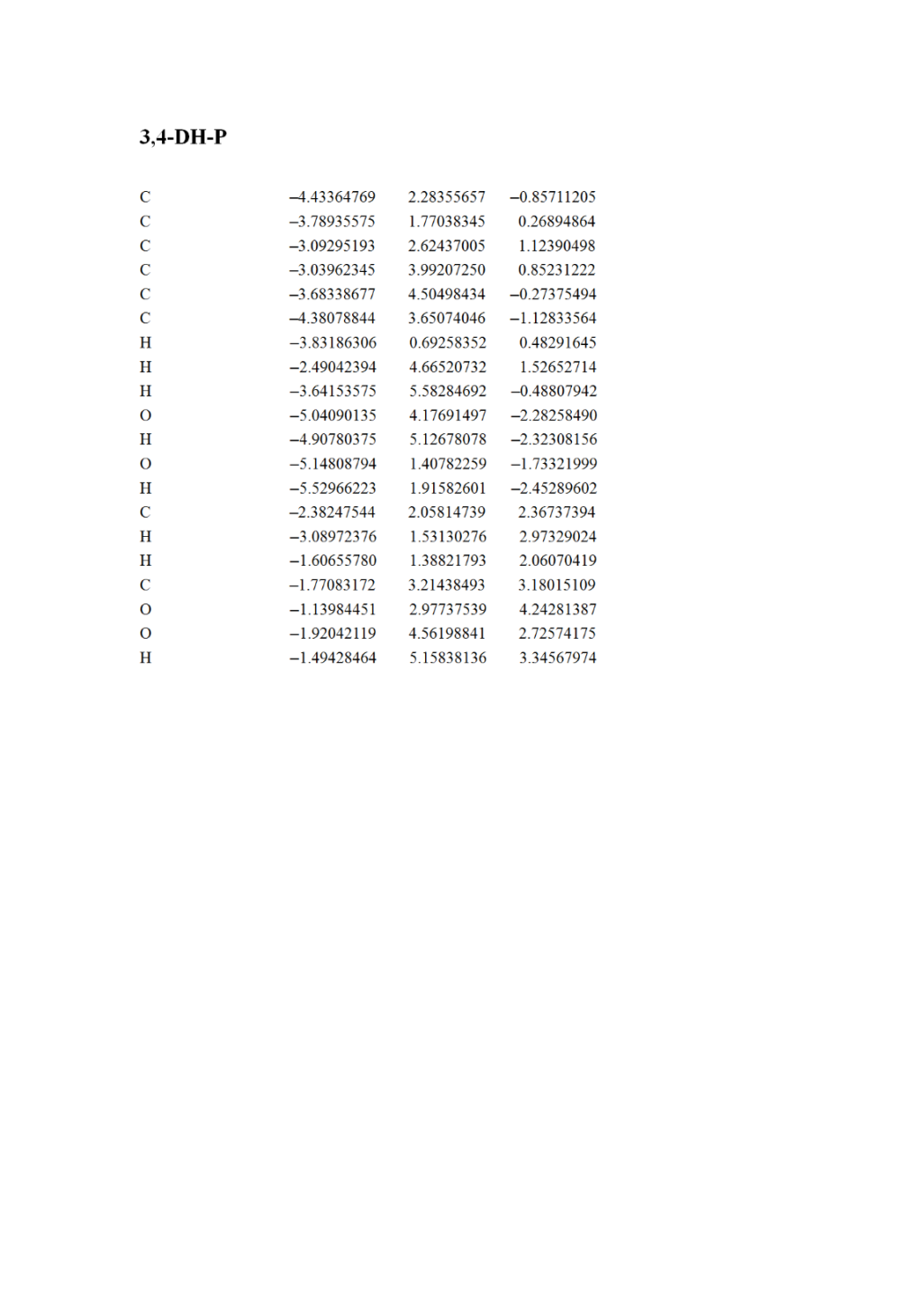


Figure S10. The Cartesian coordinates for 3,4-DH-P used in this study.


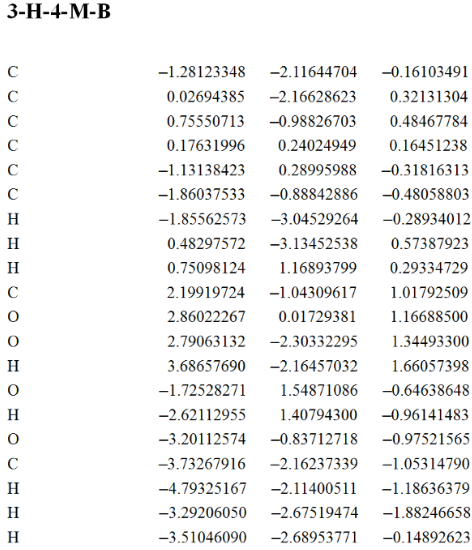


Figure S11. The Cartesian coordinates for 3-H-4-M-B used in this study.


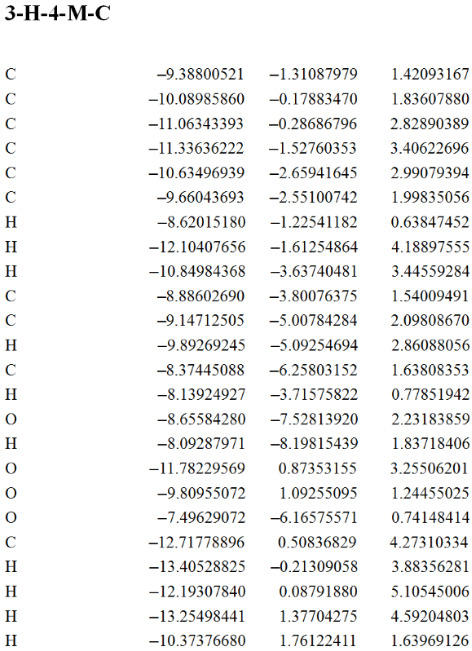


Figure S12. The Cartesian coordinates for 3-H-4-M-C used in this study.


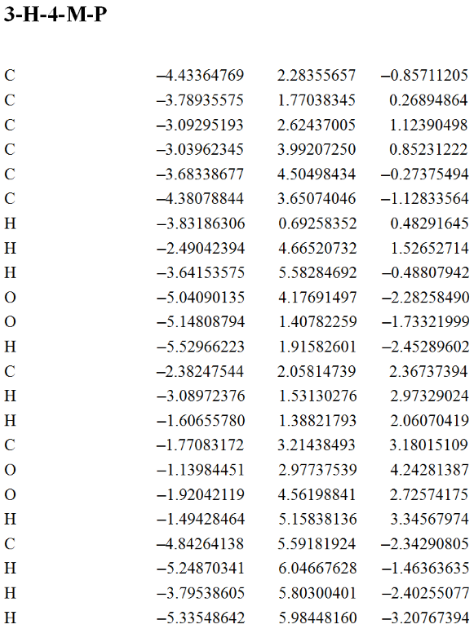


Figure S13. The Cartesian coordinates for 3-H-4-M-P used in this study.


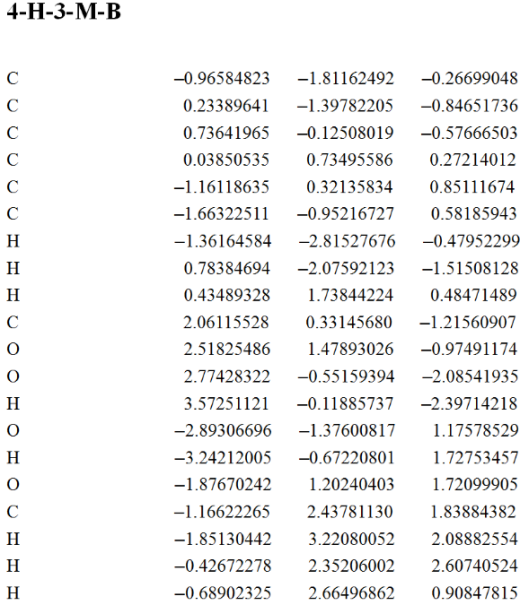


Figure S14. The Cartesian coordinates for 4-H-3-M-B used in this study.


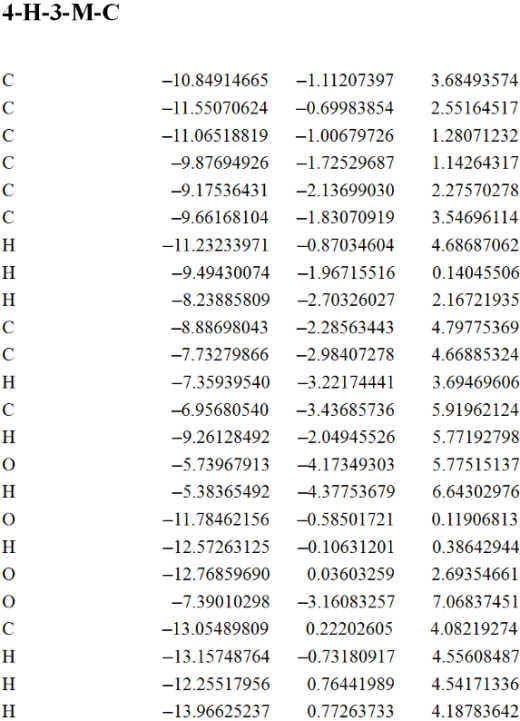


Figure S15. The Cartesian coordinates for 4-H-3-M-C used in this study.


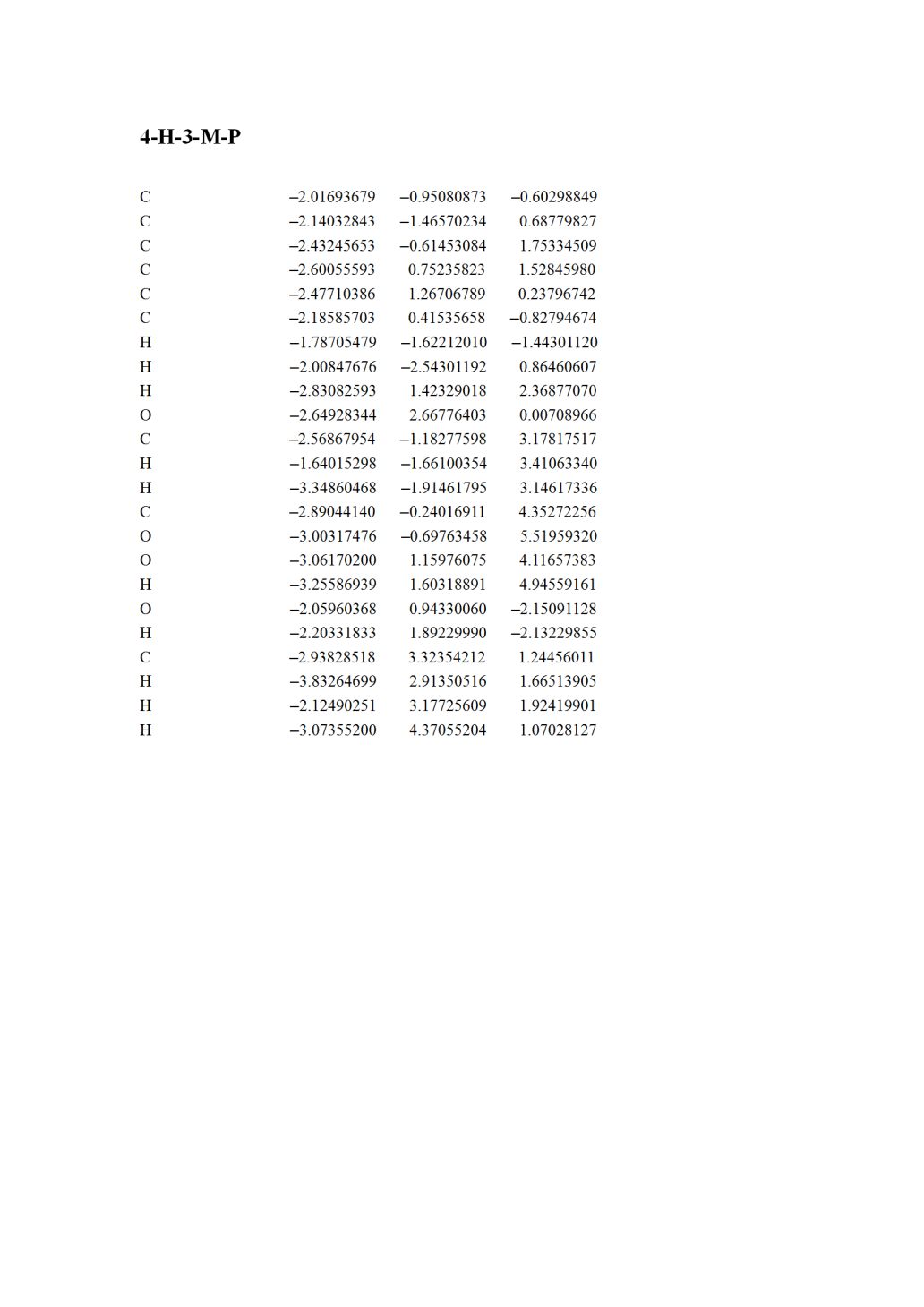


Figure S16. The Cartesian coordinates for 4-H-3-M-P used in this study.


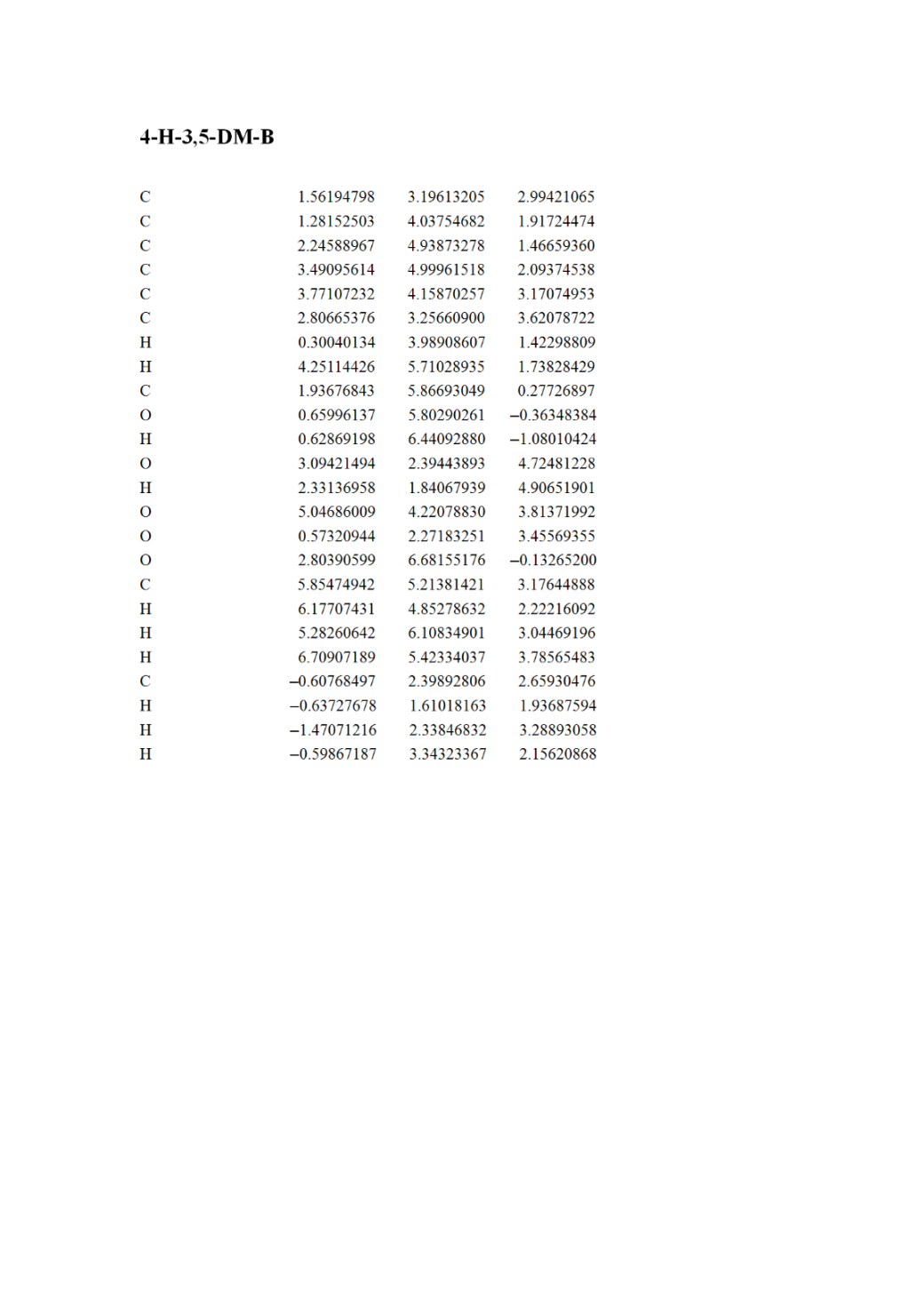


Figure S17. The Cartesian coordinates for 4-H-3,5-DM-B used in this study.


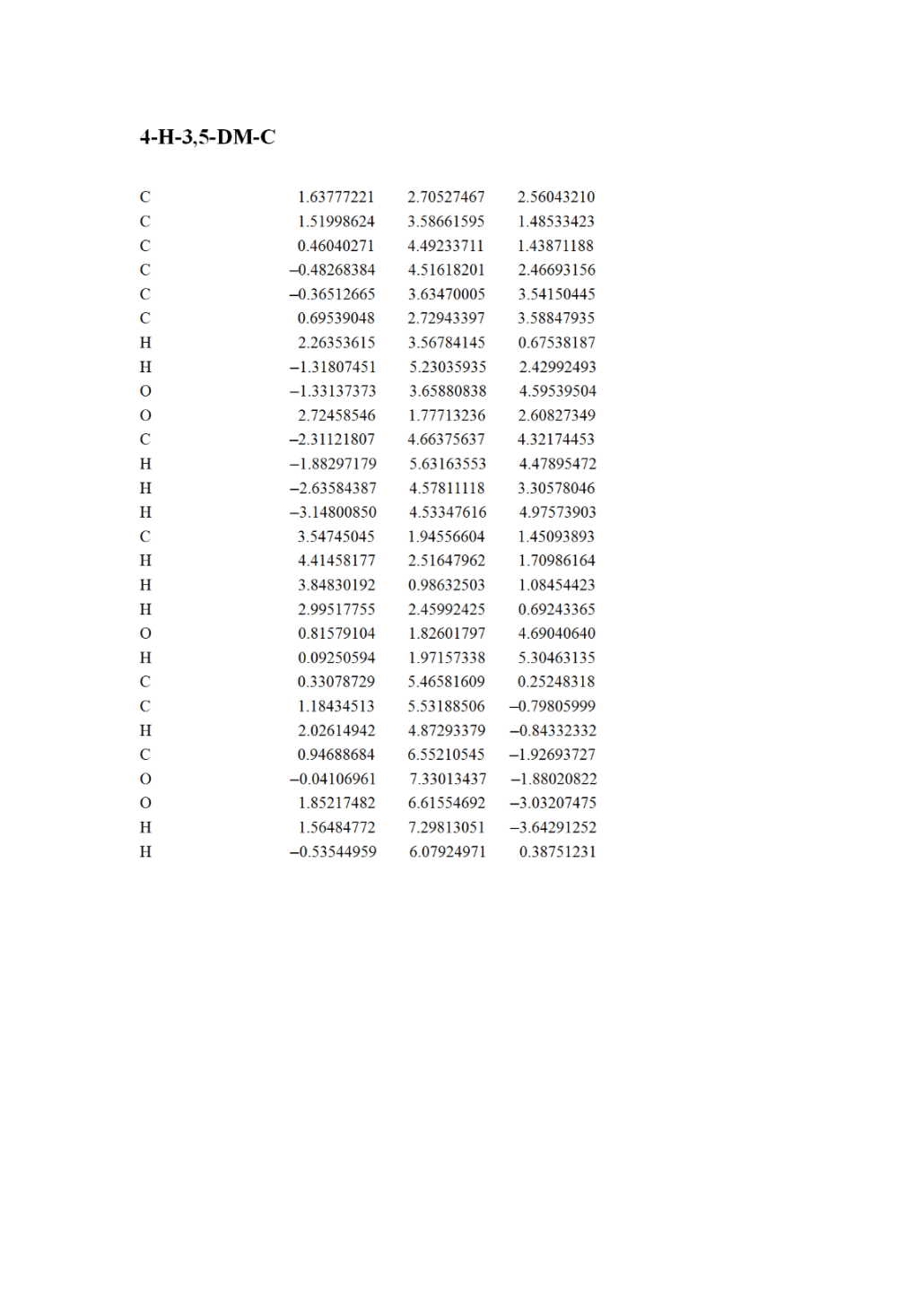


Figure S18. The Cartesian coordinates for 4-H-3,5-DM-C used in this study.


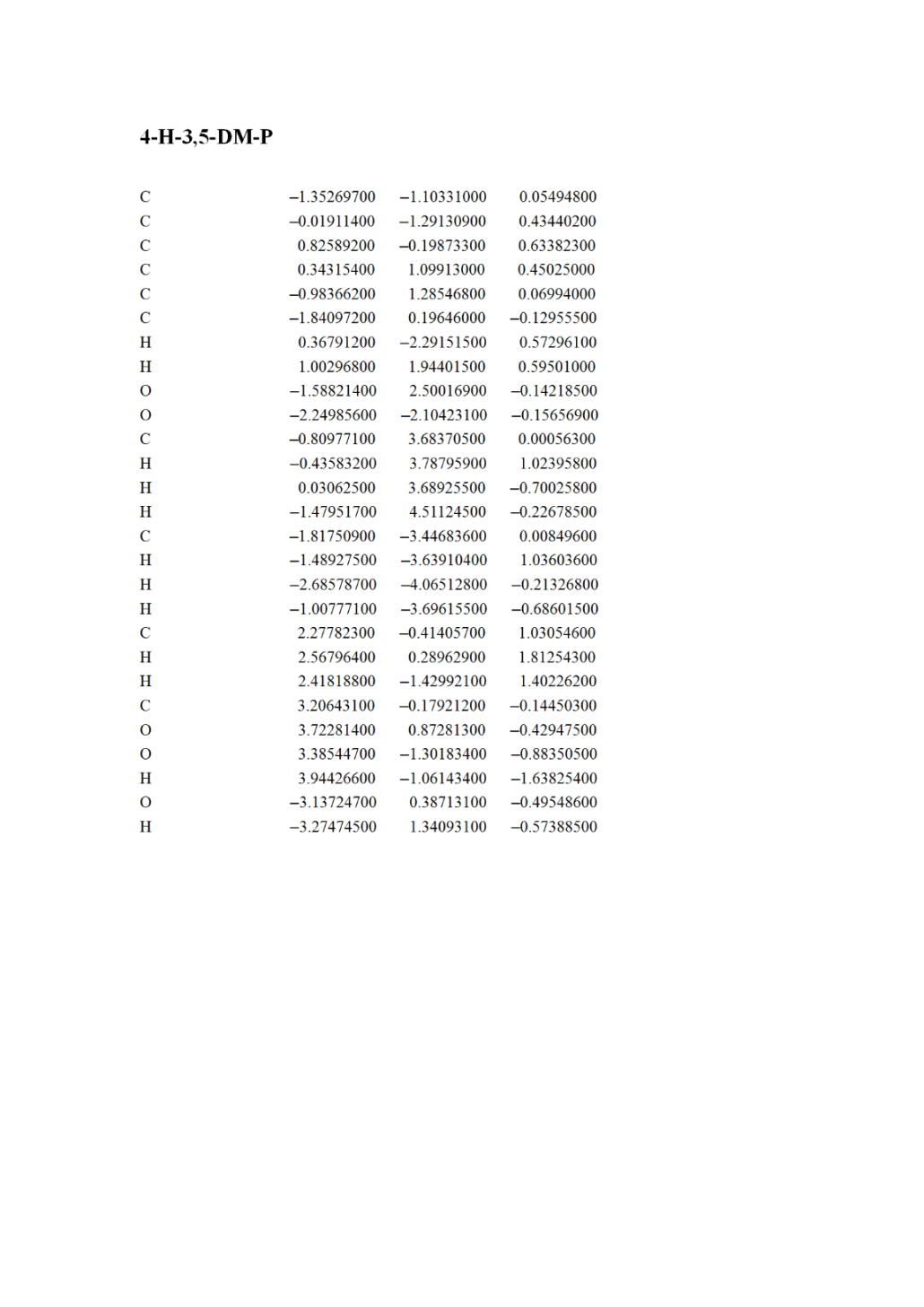


Figure S19. The Cartesian coordinates for 4-H-3,5-DM-P used in this study.
